# Virtual Patients and Sensitivity Analysis of the Guyton Model of Blood Pressure Regulation: Towards Individualized Models of Whole-Body Physiology

**DOI:** 10.1371/journal.pcbi.1002571

**Published:** 2012-06-28

**Authors:** Robert Moss, Thibault Grosse, Ivanny Marchant, Nathalie Lassau, François Gueyffier, S. Randall Thomas

**Affiliations:** 1 IR4M UMR8081 CNRS, Université Paris-Sud, Orsay, France; 2 Institut Gustave Roussy, Villejuif, France; 3 Melbourne School of Population Health, The University of Melbourne, Melbourne, Australia; 4 Escuela de Medicina, Departamento de Pre-clínicas, Universidad de Valparaíso, Valparaíso, Chile; 5 IMTh – Institute for Theoretical Medicine, Lyon, France; 6 Université Lyon 1, CNRS, UMR 5558, Laboratoire de Biométrie et Biologie Evolutive, Lyon, France; 7 INSERM, CIC 201, EPICIME, Lyon, France; 8 Service de Pharmacologie Clinique, Hop L Pradel, Centre Hospitalier Universitaire Lyon, Lyon, France; Medical College of Wisconsin, United States of America

## Abstract

Mathematical models that integrate multi-scale physiological data can offer insight into physiological and pathophysiological function, and may eventually assist in individualized predictive medicine. We present a methodology for performing systematic analyses of multi-parameter interactions in such complex, multi-scale models. Human physiology models are often based on or inspired by Arthur Guyton's whole-body circulatory regulation model. Despite the significance of this model, it has not been the subject of a systematic and comprehensive sensitivity study. Therefore, we use this model as a case study for our methodology. Our analysis of the Guyton model reveals how the multitude of model parameters combine to affect the model dynamics, and how interesting combinations of parameters may be identified. It also includes a “virtual population” from which “virtual individuals” can be chosen, on the basis of exhibiting conditions similar to those of a real-world patient. This lays the groundwork for using the Guyton model for *in silico* exploration of pathophysiological states and treatment strategies. The results presented here illustrate several potential uses for the entire dataset of sensitivity results and the “virtual individuals” that we have generated, which are included in the supplementary material. More generally, the presented methodology is applicable to modern, more complex multi-scale physiological models.

## Author Summary

As our understanding of the human body at all scales increases, the construction of a “Virtual Physiological Human” is becoming more feasible and will be an important step towards individualized diagnosis and treatment. As computational models increase in complexity to reflect this growth in understanding, analysis of these models becomes ever more complex. We present a methodology for systematically analysing the interactions between parameters and outputs of such complicated models, using the Guyton model of circulatory regulation as a case study. This model remains a landmark achievement that contributed to the development of our current understanding of blood pressure control, and we present the first comprehensive sensitivity analysis of this model. Effects of varying each parameter are explored over randomized simulations; our analysis demonstrates how to use these results to infer relationships between model parameters and the predicted physiological behaviour. Understanding these relationships is of the utmost importance for developing an optimal treatment strategy for individual patients. These results provide new insight into the multi-level interactions in the cardiovascular-renal system and will be useful to researchers wishing to use the model in pathophysiological or pharmacological settings. This methodology is applicable to current and future physiological models of arbitrary complexity.

## Introduction

Global initiatives such as the IUPS Physiome project [1], [2] and the Virtual Physiological Human (VPH) project [3], [4] aim to quantitatively understand human physiology at all levels from gene to organism through the use of mathematical modelling. Beyond a certain degree of complexity, the combinatorial number of interactions between the parts of a system can defy intuition and present severe challenges [5]. Mathematical models are appropriate tools for developing our understanding of human physiology, since they can be used to represent and analyse the combinatorial number of interactions between parameters in a rigorous and systematic manner [6].

In short, computational models that integrate physiological data from multiple scales (both physical and temporal) provide a framework for understanding the maintenance of biological entities under physiological and pathological conditions. One significant application for such models is *individualized predictive* medicine; i. e., tailoring models to the characteristics of an individual patient and predicting the outcomes of different treatment strategies, to help select the best strategy for that patient [3].

Many challenges must be overcome before a truly integrative model of human physiology can be constructed [6], [7]. Gaining a real quantitative understanding of the phenotypic variation in humans as a function of genes and environment in a mechanistic sense (i. e., understanding the genotype-phenotype map in both the explanatory and predictive sense [8]–[10]) is a tremendous challenge that awaits technological, conceptual and methodological breakthroughs [11].

A number of models have already been used to develop insight into aspects of human physiology [12]–[22], many of which have their origin in the control-theory model of whole-body circulatory regulation introduced by Guyton et al. in 1972 [23], [24]. Although it was published over 30 years ago, the Guyton model remains a landmark achievement, and with the rise in the last 10 years of systems physiology, it has attracted renewed attention [18], [25]–[27] and even generated some recent controversy [24], [28]–[30]. It was the first “whole-body”, integrated mathematical model of a physiological system; it was particularly instrumental in identifying and exploring the relationship between blood pressure and sodium balance, and in demonstrating the key role of the kidney in long-term regulation of blood pressure. It allows for the dynamic simulation of systemic circulation, arterial pressure, and body fluid regulation, including short- and long-term regulations.

In previous work, the Guyton models were modularized and re-implemented in Fortran, C++ (M2SL [31]), and Simulink [14]. Furthermore, since one of the main limitations of the early Guyton models is the low-resolution description of most of their constituting modules, a framework was built to allow replacement of the original sub-modules by new versions at a higher temporal or spatial resolution [32]; e. g., a pulsatile heart was introduced to treat systolic and diastolic blood pressures instead of only mean blood pressure [33], and a detailed model of the renin-angiotensin-aldosterone system (RAAS) has also been integrated [34]. That work was also linked to efforts in the European VPH via two Exemplar Projects, one of which used our modular reimplementation of the Guyton model as the basic set of “bricks” for a collaborative core-modeling environment for multi-organ physiology modeling [13], [14], and the other uses the Guyton model as a demonstrator for the tagging of parameters and variables with a set of reference ontologies common to databases of high-throughput genomic and proteomic data [35]. Collaborators in the Physiome/VPH community have also carried out XML markup of the individual modules of the Guyton model in CellML (http://models.cellml.org/workspace/guyton_2008), thus providing precious documentation of its structure and content.

The analysis and results presented here arose naturally from this body of work. Our motivation was to develop a methodology for systematically exploring the ramified implications of multi-parameter interactions in multi-scale physiological models. We present such a methodology, which incorporates the *elementary effects* technique introduced by Morris [36]. As a case study, we present a sensitivity analysis of the 1992 version of the Guyton model [24], [30], [37], with a focus on the multiple interactions involved in blood pressure regulation. This version was never published, but represents a more complete and modern understanding of the cardiovascular system [24], [30] (e. g., the inclusion of ANP [37]), and it is the version that members of the Guyton group have continued to use. Indeed, such a model, grounded in decades of hands-on experimental work and built with an engineer's approach to control processes, should serve as a rigourous platform for discovery of non-intuitively obvious relationships. However, despite the significance of the Guyton model, the dynamics of the model have not yet been analysed in a systematic and comprehensive study.

The results provide valuable information about the inter-dependencies of parameter effects on the model outputs, thus providing direction for future physiologically-applicable sensitivity studies of the effects of changes to multiple parameters. These results also lay the groundwork for the use of multi-parameter models such as the Guyton model in systematic *in silico* exploration of possible new drug effects, hypotheses about multiple perturbations leading to disease states, and alternative treatment strategies.

An additional outcome is the production of a *virtual population*, where each *virtual individual* is characterized by its set of parameter values (loosely analogous to genotypes) and the associated outputs (“phenotypes”). Note that the parameters of the Guyton model are in fact lower-level phenotypes, but as models continue to span larger physical and temporal scales, model parameters will approach the genotype level [38], [39]. A given real-world patient can be associated with one or more of these virtual individuals on the basis of clinically identifiable parameters or dynamics (e. g., mean arterial pressure, serum total protein, cardiac output, heart rate). Searching an existing collection of simulations in this manner avoids the inherent pitfalls in solving the inverse problem of (uniquely) identifying unknown model parameters and states from clinical observations [40]. Thus, the construction of a comprehensive virtual population could prove a useful tool in future efforts to provide efficient, individualized health-care.

Note that beyond the methodology itself, the results presented in this manuscript also serve to demonstrate some of the uses to which the complete set of elementary effects and virtual individuals may be applied. We provide tables of all of the resulting output in the supplementary material (Dataset S1), which we hope will be of use in physiological, pathophysiological and clinical settings.

## Methods

### Elementary effects

The Guyton model comprises 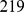 parameters and 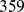 output variables. We restricted our analysis to 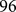 parameters {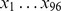} and 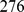 output variables {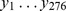} (as indicated in Equation 1, Table 1, and documented in Tables S1 and S2), focusing on those parameters with direct physiological relevance and ignoring parameters with no clear physiological interpretation (such as curve-fitting coefficients). The distribution of these 96 parameters was: 32 cardiac, 21 renal, 16 autoregulation, 16 hormonal, 11 local circulation, and 4 thirst-related. To determine which parameters have significant effects on each of the model outputs, we computed the *elementary effects* of each parameter using a modification of the formula defined by Morris [36], which we now detail.

**Table 1:** Equations for calculating elementary effects.

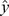 =	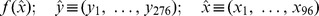	(1)
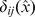 =	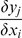	(2)
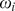 =	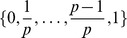	(3)
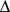 =	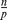	(4)
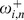 =	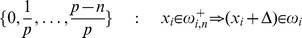	(5)
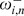 =	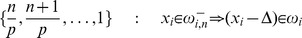	(6)
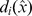 =	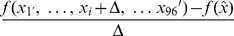	(7)
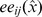 =	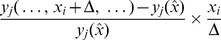	(8)

The influence 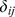 of the parameter 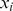 on some output 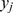 is defined by Equation 2. Assuming that each parameter 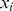 is normalized to the unit interval that 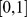, the region of experimentation––the portion of the parameter space that will be explored––is a regular 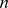-dimensional 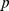-level grid 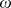, where each parameter 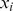 may take on values from 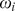 (Equation 3). For each parameter 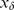 in turn, a perturbation 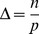 is chosen (Equation 4). For positive perturbations 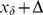, we restrict 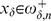 (Equation 5), and for negative perturbations 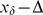, we restrict 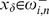 (Equation 6), so that 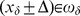.

For any point 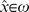 (where 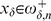 or 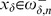), Morris defined the *elementary effect* 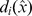 of 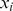 as per Equation 7. In our analysis of the Guyton model, we chose to normalize the elementary effects with respect to 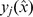 (Equation 8) rather than by 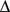, which is always a fixed percentage of the range of 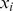.

Each elementary effect was calculated 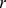 times, where each of the 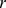 simulations was performed with randomized values for all parameters 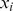, in order to obtain a representative sample of the magnitude of the effect.

Given a set of values for a single elementary effect 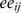, it is important to note that the mean and variance of this set provide different insights into the nature of the relationship between the parameter 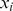 and the output 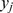. The mean indicates the *sensitivity* of 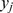 to 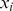, while the variance indicates the *influence of other parameters* on this relationship or the non-linearity of the effect.

### Monte Carlo simulations

For each random input vector 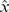, a simulation was started with the default initial state (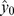) and progressed for four weeks of simulation (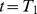), at which time a pseudo-steady state had either been reached, or a new random input vector 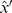 was chosen and the simulation was restarted.

The parameter under investigation (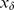) was then incremented (or decremented) by 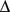 and the simulation continued for another four weeks of simulation time, after which either a new pseudo-steady state had been reached, or a new random input vector 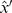 was generated and the simulation was restarted.

Throughout the simulations, a number of output variables were monitored to ensure that they remained within physiological bounds (i. e., that the virtual individuals remained “alive”, see Table 2). If these bounds were violated during a simulation, the simulation was discarded and a new input vector 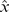 was chosen.

**Table 2:** The conditions on various model parameters that were used to ensure that the *virtual person* remained “alive” during a simulation, based on definitions from the Common Terminology Criteria for Adverse Events v4.03 (CTCAE) [41].

Parameter	Minimum	Maximum	Unit
GFR	0.015	–	L/min
CNA	120	160	mEq/L
CKE	2.5	8	mEq/L
HM	24	80	–
MAP	50	200	mmHg
HR	20	200	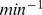

Since the system is highly non-linear, the effects of a perturbation in the parameter 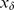 on the output variables 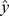 vary over time, so elementary effects were calculated at times 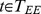 (Equation 9) and the state of the model (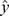) was recorded at times 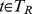 (Equation 10). The parameters for this mass-simulation process are given in Table 3.

**Table 3:** Parameters for building the virtual population.

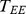 =	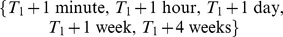	(9)
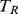 =	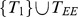	(10)
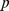 =	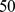	(11)
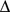 =	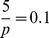	(12)
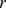 =	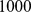	(13)
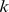 =	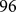	(14)

This entailed 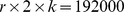 simulations to obtain 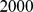 estimates (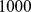 with positive perturbations and 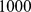 with negative perturbations) of the elementary effect of each parameter on each output. In each simulation, two distinct points in parameter space (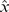 before and after the perturbation) resulted in two steady states. Each input vector and steady state can be viewed as a *virtual individual*; that is, a virtual human whose “genotype” is described by the input vector and whose “phenotype” is described by the resulting steady-state outputs. Thus, the sensitivity analysis simulations also produced a *virtual population* of 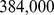 virtual individuals. We detail how this virtual population may be of use for diagnosis and exploration of treatment strategies for real-world patients in our discussion.

## Results

The results presented here are intended as a demonstration of the analyses that are possible with the complete set of simulation results, which are given in the supplementary material, namely: means and deviations of each elementary effect at each time 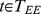; correlations between each parameter and each variable at each time 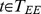 and at time 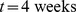 (steady-state) for both the normotensive and hypertensive sub-populations; and correlations between each elementary effect and each variable at all times 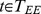.

**Figure 1:**
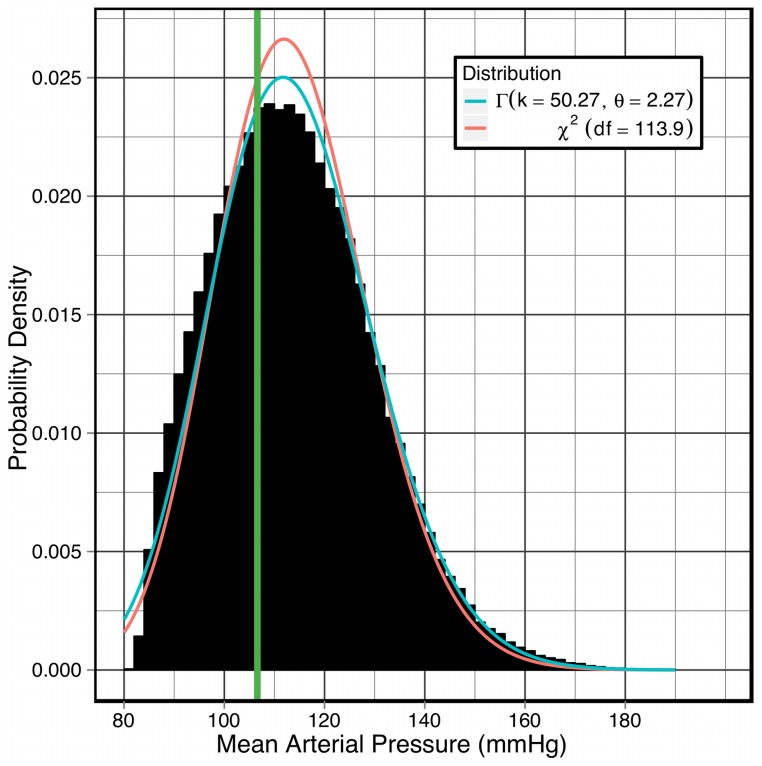
Probability density of mean arterial pressure (MAP) in the virtual population. The vertical line marks the threshold of hypertension (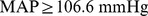), and both gamma and chi-squared distributions have been fitted.

The distribution of mean arterial pressure (MAP) in the virtual population is shown in Figure 1. Given the Common Terminology Criteria for Adverse Events v4.03 (CTCAE) [41] definition of Stage 1 hypertension (systolic BP 140–159 mmHg or diastolic BP 90–99 mmHg) and the formula for estimating mean arterial pressure from systolic and diastolic pressures (
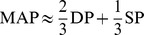
 [42]), we define hypertensive individuals as those with 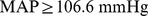. Approximately one third of the virtual individuals were normotensive and two-thirds were hypertensive (see Table 4; using an older definition of hypertension (160/95) leads to 41% of virtual individuals being classified as hypertensive). These proportions differ by less than 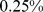 in the pre-perturbation and post-perturbation steady states, and near-identical proportions were also observed in earlier sets of simulations (not presented here). This demonstrates that the prevalence of hypertension in the virtual population is *robust* and not dependent on the choice of random input vectors 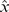.

**Table 4:** Categorization of the virtual individuals into normotensive and hypertensive populations, based on mean arterial pressure (MAP).

Population	Criteria	Size	Fraction
Normotensive	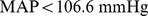	135,263	35%
Hypertensive	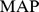 ≥ 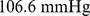	248,737	65%

Also shown in Figure 1 are gamma and chi-squared distributions that have been fitted to the probability density. The chi-square distribution is a special case of the gamma distribution where the *scale* parameter is 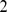. While the distributions provide reasonable fits, they both underestimate the density for 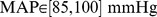 and overestimate the density for 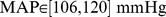.

The analysis of the 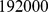 simulations investigated several aspects of the resulting data. First, we present the sensitivity analysis of the elementary effects on key output variables. The purpose was to determine which parameters induced consistent effects when perturbed, and how these effects are influenced by other parameters. Second, the correlations between parameters and key variables were considered, to identify relationships between the outputs and fixed parameter values. Note that while the elementary effects are shown to vary over time, the correlations remained essentially constant. These correlations were then compared across the normotensive and hypertensive sub-populations, to detect any differences in these relationships between these two populations. Finally, several generalized linear models (GLMs) [43], [44] were evaluated for their predictive power of identifying hypertensive individuals based on a select number of parameters.

Definitions of all model 96 parameters and 276 variables are tabulated Tables S1 and S2. More complete results are tabulated in Dataset S1.

### Sensitivity analysis

**Figure 2:**
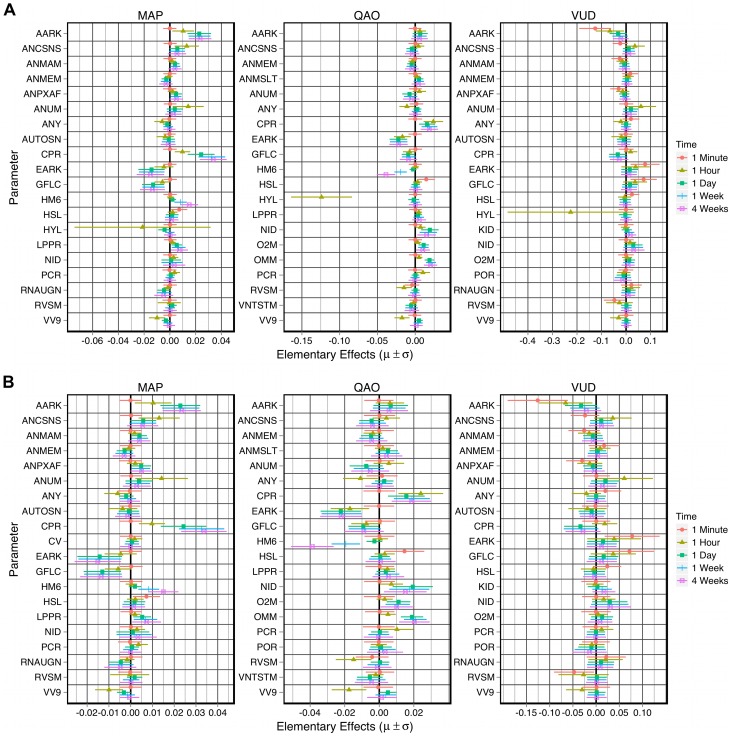
The most significant elementary effects on three key output variables at each time 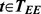. **A:** The effects on mean arterial pressure (MAP), cardiac output (QAO) and rate of urine production (VUD) are plotted (at 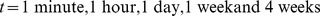 after the perturbations) as 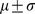. **B:** The most significant elementary effects when HYL is ignored. The complete tables of elementary effects are included in the supplementary material.

Given our interest in the development of hypertension, we focus the discussion here on variables directly related to blood pressure. For example, Figure 2a shows the most significant elementary effects (at each time 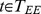) on three such variables: the mean arterial pressure (MAP), the cardiac output (QAO), and the rate of urine production (VUD).

**Figure 3:**
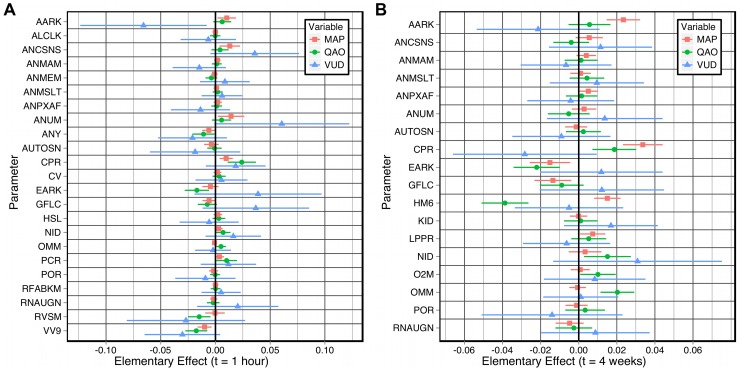
The largest elementary effects on three key output variables at 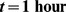 and 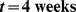. The effects on mean arterial pressure (MAP), cardiac output (QAO) and rate of urine production (VUD) are plotted as 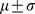. Elementary effects were sorted by the magnitude of their largest effect on the three output variables. **A:** The elementary effects at 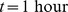 (excluding HYL). **B:** The elementary effects at 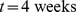.

The single largest effect on all three variables is that of HYL (the quantity of interstitial hyaluronic acid), which affects the tissue hydrostatic and osmotic pressures. This effect is only observed *one hour after* the perturbation is made. That is, a change in hyaluronic acid takes more than one minute to have an effect, and the effect is no longer evident after 24 hours. The large deviations (significantly larger than those of any other parameter) demonstrate that the effects of HYL are highly non-linear. We will demonstrate how to identify interesting multi-parameter effects, using HYL as an example. To clearly depict the other elementary effects, they are shown in Figure 2b without the effects of HYL. The largest steady-state elementary effects at 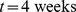 are shown in Figure 3. The complete table of elementary effects is available in the supplementary material.

#### Effects on mean arterial pressure (MAP)

Consider the elementary effects on MAP at time 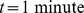. The only appreciable effect is that of HSL (the basic strength of the left ventricle). This (comparatively small) effect is not accompanied by an effect of HSR (the basic strength of the right ventricle). The effect of HSL at times 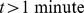 were negligible.

At time 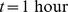, AARK (the basic resistance of the afferent arteriole) has an effect on MAP, as do ANCSNS (sensitivity controller, general angiotensin effect), ANUM (sensitivity controller, angiotensin effect on arterial resistance and venous volume), ANY (sensitivity controller, angiotensin effect on venous volume), CPR (the critical plasma protein concentration for protein destruction), EARK (the basic resistance of the efferent arteriole), GFLC (glomerular filtration coefficient) and VV9 (basic venous volume).

Some of these effects wane over time (ANCSNS, ANUM, ANY and VV9), while the remaining effects become stronger over time (AARK, CPR, EARK, GFLC) and exhibit the largest steady-state elementary effects on MAP. Other parameters also exhibit significant elementary effects over the longer timescales: HM6 (erythropoietic limiter), LPPR (rate of liver protein production), NID (rate of sodium intake) and RNAUGN (basal renal autoregulation feedback multiplier).

Thus, as the model approaches the steady-state following a perturbation, the effects of hormones such as angiotensin are reduced, whilst properties that directly affect glomerular filtration exhibit the largest elementary effects. As one would expect, an increase in AARK or a decrease in EARK (in isolation) results in a permanent increase in the mean arterial pressure, due to a decreased filtration rate. A decrease in glomerular permeability (GFLC) produces a similar change for the same reason. The other major long-term effects (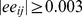) are exerted by ANCSNS, ANMAM (sensitivity of afferent arterioles to angiotensin), ANPXAF (sensitivity of afferent arteriole resistance to ANP), CPR, HM6 and LPPR. The first three parameters affect vasoconstriction in general (ANCSNS) and in the afferent arteriole (ANMAM, ANPXAF), while the latter three affect the plasma protein concentration and hematocrit (CPR, HM6, LPPR) and thus affect the driving pressure gradient for glomerular filtration. Note that the elementary effects of these parameters are not evident until 1 day or 1 week after the original perturbation. These observations reflect the infinite gain of the kidney in long-term regulation of arterial blood pressure [45], [46], as originally proposed by Guyton [23].

#### Effects on cardiac output (QAO)

The only observable effects on QAO at time 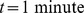 are from HSL and RVSM (basal systemic venous resistance multiplier). As was shown for the elementary effects on MAP, HSL exerts a short-term elementary effect on QAO, and there is no elementary effect from HSR.

A number of parameters exhibit significant effects at time 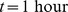 that do not persist over longer timescales: ANY, PCR (critical capillary pressure for protein leakage), RVSM and VV9. Other parameters begin to exhibit elementary effects on QAO at time 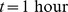 or 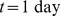, which then persist in the steady-state: AARK, ANUM, CPR, EARK, GFLC, NID, O2M (basic 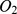 utilization in non-muscle tissue) and OMM (basic 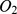 utilization in muscle tissue, at rest). The most significant permanent effect on QAO is exerted by HM6, the magnitude of which greatly increases from 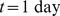 to 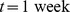 to 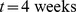.

The major long-term effects on cardiac output (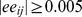) govern: plasma protein concentration and hematocrit (CPR, LPPR, HM6), the effect of angiotensin on arterial resistance and venous volume (ANUM), glomerular filtration (AARK, EARK, GFLC), sodium intake (NID) and 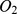 utilization (O2M, OMM).

The nature of the steady-state effects on QAO differ from those on MAP. Several parameters cause permanent changes in both QAO and MAP, but with smaller effects on QAO: AARK (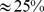), CPR (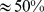) and GFLC (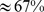). Conversely, perturbations in EARK and NID have larger long-term effects on QAO than on MAP (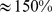 and 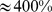, respectively). One parameter, HM6, exerts contrary effects on MAP and QAO; an increase in HM6 causes an increase in arterial pressure but decreases the cardiac output with an effect approximately 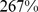 greater than the effect on MAP. An increase in oxygen utilization in either muscle or non-muscle tissue (OMM or O2M) results in a raised cardiac output to increase the oxygen supply, but does not exert an elementary effect on the mean arterial pressure. However, an increase in the rate of protein production in the liver (LPPR) exerts near-identical effects on the long-term mean arterial pressure and the cardiac output.

#### Effects on urine production (VUD)

The elementary effects on VUD at time 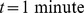 are from: AARK, ANCSNS, ANY, EARK, GFLC, RNAUGN, RVSM, ANMAM, ANMEM (sensitivity of efferent arterioles to angiotensin) and ANPXAF. Of these parameters, the effects of AARK, ANCSNS, EARK, GFLC and RNAUGN greatly diminish over time, and the effects of ANY, RVSM, ANMAM, ANMEM and ANPXAF effectively disappear by the time the model has reached the steady-state (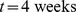). This is consistent with experimental observations that urine production is rapidly altered in response to these perturbations [47]–[49] and serves to maintain the arterial pressure [45], [50], but that the bulk of the change is transient (see Figure 3). This response is mediated, at least in part, by activity of the renal sympathetic nerves [51], [52].

Several parameters that exhibited no elementary effect at 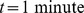 can be seen to exert an effect at 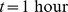: ANUM, AUTOSN (sensitivity controller, overall non-muscle vascular resistance autoregulation), CPR, NID, POR (the reference value of capillary 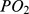 in non-muscle tissue) and VV9. The effects of ANUM, AUTOSN and VV9 wane over longer timescales (VV9 exerts no effect at all by the steady-state), but the effects of CPR, NID and POR are persistent. Note that the initial effect of CPR is positive (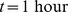), but that the effect is reversed at all subsequent times.

By time 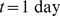, the elementary effects of all parameters have essentially converged to their steady-state values. The major long-term effects on urine production (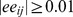) are exerted by parameters that have direct control over the glomerular filtration (AARK, EARK, GFLC), the effects of angiotensin (ANCSNS, ANUM), plasma protein concentration (CPR), sodium and potassium intake (KID, NID) and non-muscle capillary 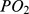 (POR, which affects vasoconstriction over short, intermediate and long-term timescales).

The nature of the steady-state effects on VUD (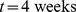) differ from those on both QAO and MAP. As can be seen in Figure 3, typically the elementary effects on VUD are significantly larger than the effects on QAO and MAP, with much larger deviations (i. e., interactions with other parameters). The same can be said of QAO in comparison to MAP. That is, urine production is more sensitive to perturbations than cardiac output, which in turn is more sensitive to perturbations than arterial pressure. This reflects Guyton's explanation that the kidney acts as a servo-controller of long-term blood pressure by adjusting salt and fluid balance [53]–[56].

#### Effects on sodium excretion (NOD)

The elementary effects on NOD (sodium excretion, not shown here) were found to be near-identical to those on VUD (urine production) at all times 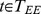. The only elementary effects that differed appreciably (by 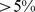) were those of KID (potassium intake), which had no effect on sodium excretion, and NID (sodium intake), which had a 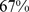 larger elementary effect on sodium excretion than on urine production.

#### Multi-parameter effects: accounting for the variance in HYL

We now demonstrate how the results of the sensitivity analysis can be used to determine which parameters influence an elementary effect. These are the parameters that are most likely to be of interest when investigating the effects of multi-parameter perturbations. By identifying such parameters with this method, the number of multi-parameter combinations under consideration can be greatly reduced, somewhat mitigating the combinatorial growth in parameters combinations as the number of model parameters increases. This information is of particular use when trying to regulate some physiological function (e. g., pharmacological applications).

As illustrated in Figures 2a and 2b, the parameter HYL exerts the greatest elementary effect on MAP, QAO and VUD at time 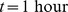. The variance of this effect is also several times larger than that of any other elementary effect, which indicates that many other parameters interact with HYL and influence this elementary effect.

**Figure 4:**
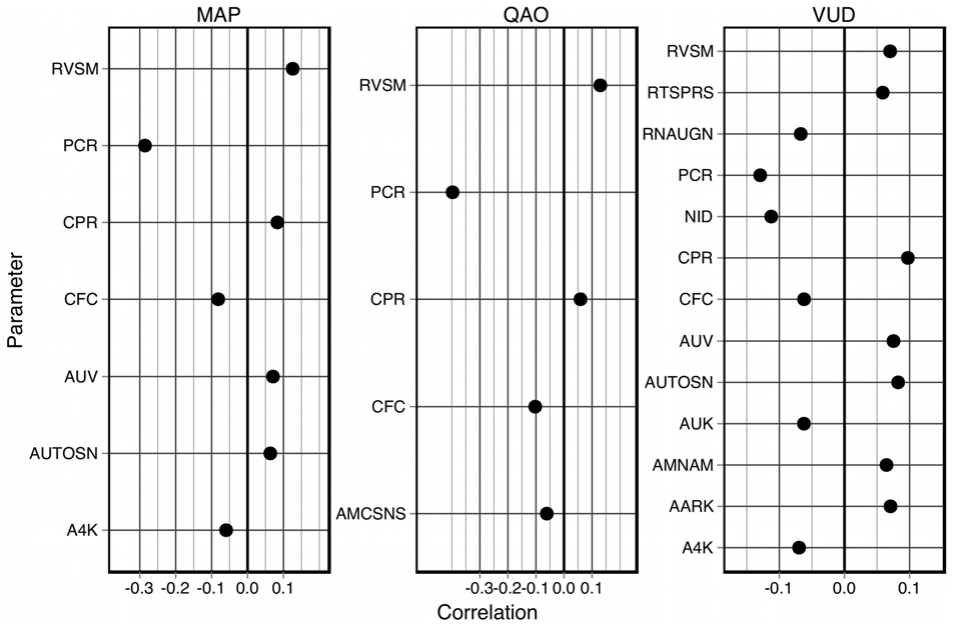
Significant correlations between model parameters and the elementary effects of HYL at 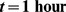. Correlations (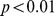) are shown for the elementary effect of HYL on mean arterial pressure (MAP), cardiac output (QAO) and rate of urine production (VUD).

The correlations and partial correlations (controlling for HYL) between the variables (MAP, QAO and VUD) and the model parameters differ by less than 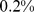 (not shown). That is, accounting for the value of HYL does not appreciably change the correlations between the variables and the other parameters. However, analysing the correlations between the *elementary effect of HYL* and the remaining 95 parameters (Figure 4) reveals that, for all three elementary effects of HYL, the most significant influence is the parameter PCR (the critical capillary pressure for protein leakage). Other parameters that are significantly correlated with the elementary effects include CFC (capillary filtration coefficient), CPR and RVSM. Sodium intake (NID) is also significantly correlated with the elementary effect of HYL on urine production.

The significant parameters that have been identified by this correlation analysis are all related to vessel and interstitial oncotic pressures, which explains the nature of their influence on the elementary effect of HYL. Hyaluronic acid plays a large role in determining the hydrostatic and oncotic pressure of the tissue gel in the Guyton model, and this effect is a function of the amount of hyaluronic acid (HYL) in the tissue and the interstitial fluid volume (VTS).

This analysis also demonstrates that the Guyton model fails to account for other physiological effects of hyaluronic acid, such as its role in water and solute balance in the inner medulla [57]–[60]. Were this effect included in the Guyton model, one would have expected a number of parameters governing renal function to be in evidence in Figure 4.

A limitation of the sensitivity analysis presented here is that only a single parameter was perturbed during each simulation. However since, for each parameter, this was done with thousands of randomized sets of values for all of the remaining parameters, we demonstrate that the results of our analysis can inform the selection of interesting/relevant multi-parameter perturbations, greatly reducing the computational cost of exhaustively searching all possible multi-parameter perturbations.

#### Summary of the elementary effects

Parameter interactions, which are evidenced by large variances, are more prevalent at the shortest time-scales (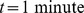 and 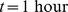) and in the largest steady-state effects (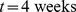), especially for the elementary effects on urine production. The results also suggest that perturbations typically exert larger effects on urine production than on mean arterial pressure and cardiac output, since at *all times* shown in Figure 2b the elementary effects on VUD are much larger than the effects on MAP and QAO at *any time*. This has been observed in animal experiments [46], [55], [61].

The parameters that demonstrated the largest elementary effects on multiple output variables at the steady-state (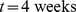) are: AARK, EARK and GLFC (renal filtration), CPR (plasma protein concentration) and HM6 (hematocrit). Parameters that govern the effects of angiotensin (ANCSNS, ANMAM, ANMSML, ANUM) exhibited smaller steady-state effects. The remaining parameters with significant steady-state effects are related to oxygen consumption (O2M, OMM), diet (KID, NID), sensitivity of afferent arteriole resistance to ANP (ANPXAF), general autoregulation (AUTOSN, RNAUGN), capillary 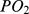 (POR) and protein production (LPPR).

A perturbation in any of these parameters changes the steady-state variable values. The importance of these parameters reflects the role of the kidney in long-term blood pressure autoregulation in both the Guyton model and human physiology [45], [46]. The effects of the parameters related to angiotensin reflect the effects of angiotensin levels on the renal function curve [54]. That the most significant renal parameters identified here (AARK, EARK and GFLC) are all directly related to *filtration* and not to tubular secretion or reabsorption is consistent with the original predictions of the Guyton model: “Under normal circumstances, renal factors that determine the glomerular filtration rate at different levels of arterial blood pressure are quantitatively more important for the control of arterial blood pressure than are renal tubular mechanisms” [53]. This also reflects an underlying limitation of the Guyton model: the renal module is highly simplistic and considers very few aspects of nephron function.

### Correlations between parameters and variables

**Figure 5:**
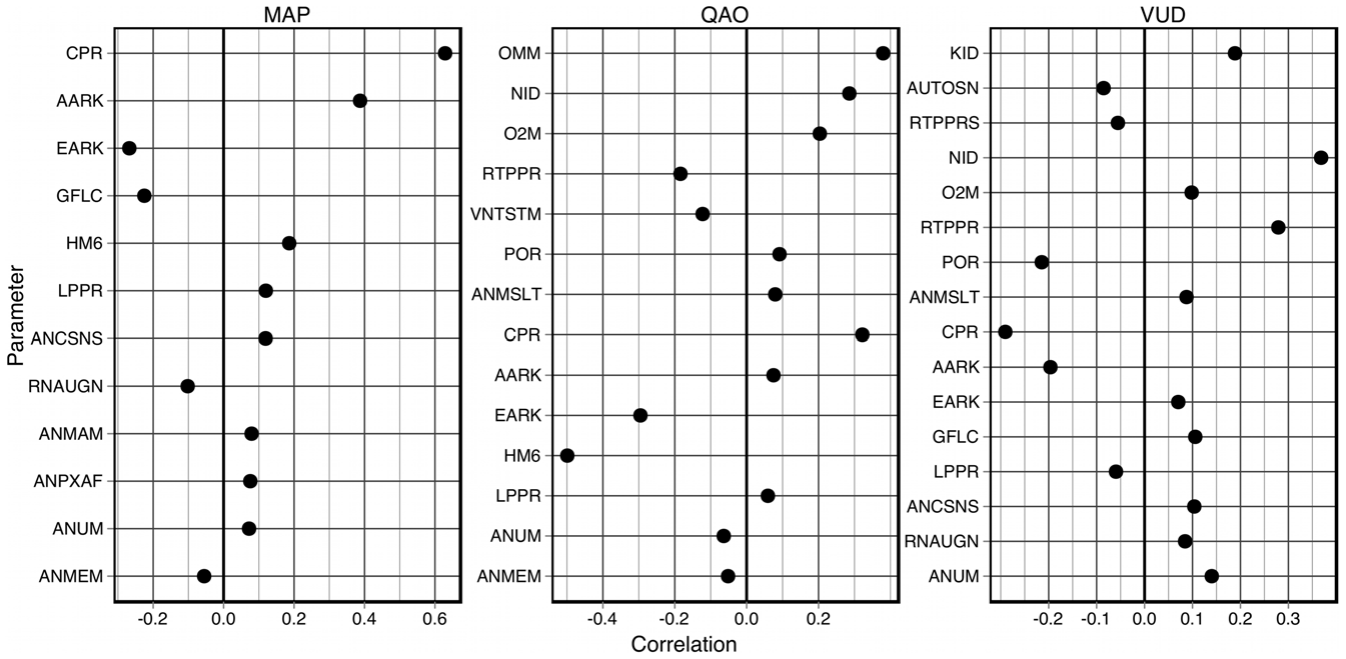
Significant correlations between model parameters and three key output variables. Correlations are shown for mean arterial pressure (MAP), cardiac output (QAO) and rate of urine production (VUD), where 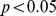 and 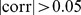.

Correlations were calculated between each parameter and each output variable at each time 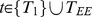, using the Spearman rank-correlation [62]. A rank-correlation method was chosen because such methods are sensitive to any near-monotonic relationship and do not assume that the data is normally distributed. The correlations showed negligible variance (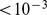) over these times, in contrast to the elementary effects presented earlier. This is because the correlations are sensitive to the *absolute value* of the parameter, while the elementary effects are sensitive to the influence of a perturbation and not the *absolute value*. Significant correlations are shown in Figure 5 for the same three variables (MAP, QAO and VUD) whose elementary effects were presented in Figure 2.

Consider the correlations with MAP; the most-highly correlated parameters (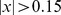) are CPR, AARK, EARK, GFLC and HM6, all of which also exhibit significant elementary effects on MAP. As noted earlier, all of these parameters affect glomerular filtration: AARK, EARK and GFLC are all related to physical properties of the glomerulus, while CPR and HM6 affect the driving pressure gradient for ultrafiltration.

In contrast, the parameters most-highly correlated with QAO (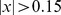) are HM6, OMM, CPR, EARK, NID, O2M and RTPPR (the effect of glomerular oncotic pressure on renal tissue oncotic pressure). RTPPR was not seen to exert a significant elementary effect on QAO, but it shows a higher correlation with QAO than do AARK, ANUM, GFLC and LPPR, all of which exerted significant steady-state effects on QAO. Three of these parameters––HM6, OMM and O2M––are directly related to oxygen supply and utilization in the body, whilst CPR and NID affect both the plasma volume and renal filtration, EARK also affects renal filtration, and RTPPR affects tubular reabsorption.

The parameters most-highly correlated with VUD (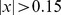) are NID, CPR, RTPPR, POR, AARK and KID. As was the case for QAO, RTPPR does not exert a significant elementary effect on VUD, but demonstrates higher correlation with VUD than do ANCSNS, ANUM, EARK and GFLC, all of which exhibit significant steady-state effects on VUD. All of these parameters, except for POR, are directly related to renal filtration and reabsorption, while POR modulates the vasoconstrictor effect on blood-flow autoregulation across rapid, intermediate and long-term timescales.

One parameter, CPR, is notable for being highly correlated with all three output variables MAP, QAO and VUD. In particular, CPR has a correlation of 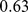 with MAP; the only other correlation greater than 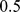 is that between HM6 and QAO (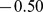). This parameter is the critical plasma protein concentration for protein destruction in the liver, which affects the colloid oncotic pressure in the vasculature. The direct effects of this parameter include the rate of glomerular filtration and the rate of capillary leakage. These observations demonstrate that the Guyton model reflects the importance of renal filtration and colloid oncotic pressure to overall haemodynamic regulation [45], [46], [54], [55].

### Normotensive vs hypertensive sub-populations

**Figure 6:**
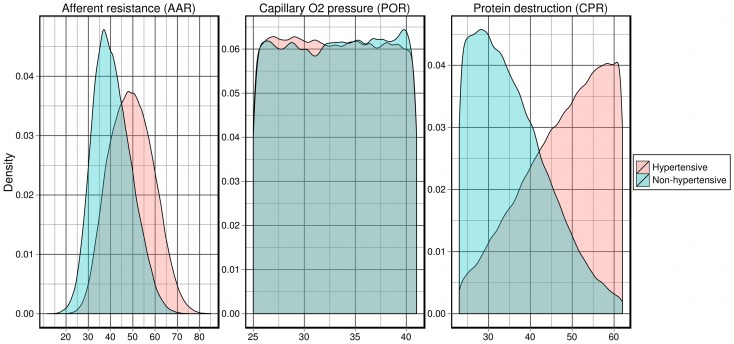
Probability densities of model variables in the normotensive and hypertensive virtual sub-populations. Probability densities are shown for AAR (the afferent arteriolar resistance), POR (the reference value of capillary 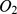 pressure in non-muscle tissue) and CPR (the critical plasma protein concentration for protein destruction).

The virtual individuals were divided into normotensive and hypertensive sub-populations based on their mean arterial pressure, as illustrated in Table 4. The probability densities of each parameter and variable were compared across these populations, as were the correlations between the model parameters and the output variables. The probability densities revealed observable differences between the populations (Figure 6), both in the model parameters (e. g., CPR) and output variables (e. g., AAR). Note that the two probability densities shown here for CPR are markedly more distinct than when CPR was classified based on the elementary effect of HYL (not shown).

However, obvious differences were observed for very few parameters, all of which had already been highlighted in the sensitivity and correlation analyses.

**Figure 7:**
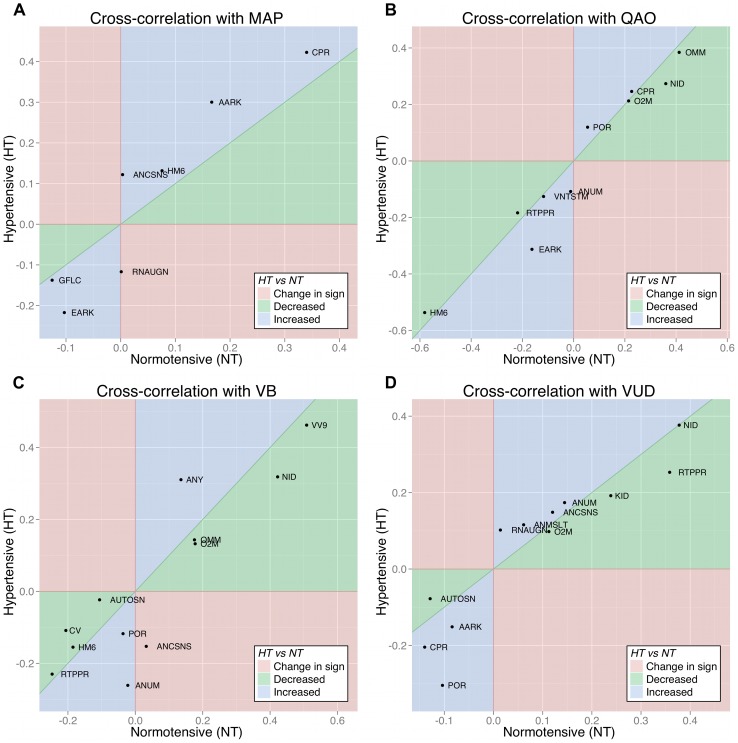
Comparison of correlations between parameters and variables in the normotensive and hypertensive virtual sub-populations. For a given variable, the correlations with each parameter are plotted against the x-axis for the normotensive population, and against the y-axis for the hypertensive population (only correlations 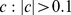 are shown). **A:** Mean arterial pressure (MAP). **B:** Cardiac output (QAO). **C:** Blood volume (VB). **D:** Rate of urine production (VUD).

Correlations between parameters and variables were then compared between the two populations; some results are shown in Figure 7. The colour-coded regions of each graph represent different relationships between the correlations: green indicates a decreased correlation in the hypertensives; blue indicates an increased correlation in the hypertensives; and red indicates that the correlation has switched sign between the two populations.

The correlations with MAP in the hypertensive population are systematically larger than those in the normotensive population (Figure 7a), which supports the notion that arterial pressure regulation has been reduced in the hypertensive population. However, the correlations with QAO show no such relationship (Figure 7b) with the sole exception of EARK. This suggests that the regulation of cardiac output has not been reduced in the hypertensive population, and that a change in cardiac output is neither a cause nor symptom of the hypertension that is observed in the virtual population, which reflects Guyton's explanation of arterial hypertension being fundamentally a renal pathology [23], [24], [56].

When correlations with blood volume are considered (Figure 7c), the parameters with the largest increases in correlation (ANCSNS, ANUM, ANY) are all related to the effects of angiotensin on arterial resistance and venous volume. Parameters with decreased correlation in the hypertensive population include NID, VV9 and CV (venous compliance). The logical inference is that angiotensin is playing a more significant role in regulating the blood volume in the hypertensive individuals than in the normotensive individuals. Angiotensin plays a role in the activation of the RAAS [56], [63], [64], which increases salt and water retention in the kidney [65]–[67] and raises the “set-point” arterial pressure that the kidney will maintain [50], and these effects are incorporated into the Guyton model. More recent studies have also revealed angiotensin's roles in hypertension via oxidative stress [68]–[70] and inflammatory vascular injury [71], [72], but these phenomena are not included in the Guyton model.

The correlations with urine production (Figure 7d) reveal changes in only a few parameters. The decreased correlation with RTPPR indicates that glomerular oncotic pressure has a smaller effect on tubular reabsorption in the hypertensive population. Of the parameters with increased correlations, AARK and POR are directly related to blood-flow autoregulation and vasoconstriction, and CPR affects the plasma colloid oncotic pressure, which affects the plasma volume and the driving pressure gradient for glomerular filtration. This leads us to conclude that the urine production in the hypertensive population is more sensitive to blood-flow autoregulation and plasma colloid oncotic pressure.

### Identifying hypertensive virtual individuals with GLMs

The large virtual population that has been assembled here (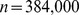) can be used not just to analyse relationships between model parameters and outputs, but also to derive and evaluate *classifiers* for predicting particular phenotypes in virtual individuals. Since hypertension places a heavy burden on health-care systems around the world, and blood pressure regulation is the chief focus of the Guyton model, the most obvious phenotype to predict is hypertension.

The virtual population was divided in two: a randomly-chosen training set 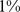 of the population size, and the remainder of the population served as an evaluation set. A generalized linear model (GLM) [43], [44] with a binomial distribution function was fitted to the training set to predict whether or not each individual was hypertensive (i. e., 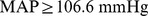). A minimal GLM was then selected by step-wise reduction of the original GLM with Akaike's information criterion (AIC) [73], resulting in a 30-parameter classifier.

**Figure 8:**
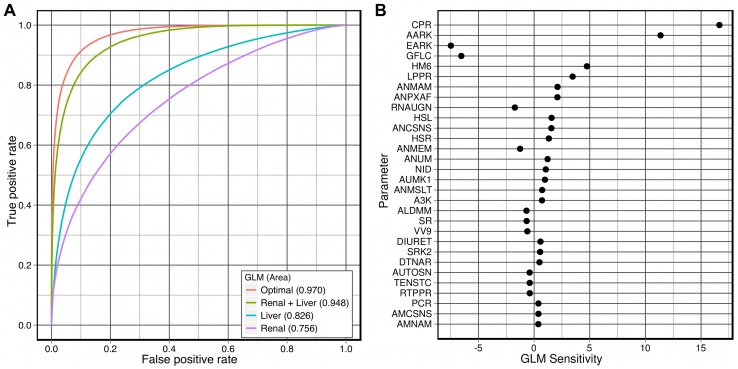
Evaluation of linear classifiers for identifying hypertensive virtual individuals. Each classifier (binomial GLM) was fitted to a random 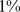 sample of the virtual population and then evaluated on the remaining 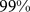. **A:** ROC curves for several classifiers; the optimal (30-parameter) classifier has an area under curve (AUC) of 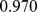, demonstrating high predictive power. The 6-parameter “Renal+Liver” classifier performs nearly as well (AUC = 0.948). **B:** The parameter sensitivity of the optimal classifier. The y-axis measures the variation in the prediction over the range of values for each parameter.

This classifier was then evaluated on the evaluation set (i. e., the rest of the virtual population), shown in Figure 8a, and demonstrated a high degree of accuracy. The sensitivity of the classifier to each of the 30 parameters is shown in Figure 8b. This list of parameters closely resembles those parameters most-highly correlated with mean arterial pressure (Figure 5).

But no matter how accurately this classifier can predict hypertension in the virtual population, one should not conclude that it will be of practical use for predicting hypertension in real-world patients. The classifier is a function of 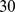 model parameters, many of which are not physiologically derived or measurable. In order to feasibly use such a classifier with real-world patients, the model parameters must be restricted to those that are readily identifiable and measurable in human beings.

Of the parameters listed in Figure 8b, we assume that CPR and LPPR can be estimated from blood tests and that the values of the renal filtration parameters AARK, EARK and GFLC could possibly be estimated from whole-body glomerular filtration rate (GFR) (or, more invasively, from a biopsy). NID can be estimated from the person's diet. The resulting classifier (“Renal+Liver” in Figure 8a, coefficients given in Table 5) predicts hypertension on the basis of these parameters (see Table 6) and suffers from a modest loss of predictive power in comparison to the optimal classifier. It can correctly identify 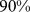 of the hypertensive virtual individuals with a 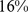 false-positive rate, in comparison to the optimal false-positive rate of 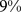. Further restricting the parameters to either solely liver-related or kidney-related (Table 6) significantly reduces the predictive power of the classifiers.

**Table 5:** The coefficients of each classifier (GLM) presented in Figure 8a.

	Classifier			
Parameter	Optimal	Renal+Liver	Liver	Renal
(intercept)	−32.66	−12.6066	−5.3854	0.92989
A3K	1.073e-5			
AARK	16.95	11.9073		4.63921
ALDMM	−0.1565			
AMCSNS	0.5476			
AMNAM	0.2681			
ANCSNS	2.312			
ANMAM	2.599			
ANMEM	−1.069			
ANMSLT	0.2056			
ANPXAF	0.5081			
ANUM	0.2067			
AUMK1	1.836			
AUTOSN	−0.9326			
CPR	0.3703	0.2589	0.1310	
DIURET	0.1422			
DTNAR	0.5744			
EARK	−7.800	−5.7318		−2.56488
GFLC	−307.4	−235.5629		−115.02363
HM6	3.451e-3			
HSL	1.315			
HSR	1.374			
LPPR	26.90	18.9798	9.7504	
NID	4.322	2.7301	−0.2501	0.07619
PCR	2.188e-2			
RNAUGN	−1.645			
RTPPR	−0.1121			
SR	−0.3817			
SRK2	3.059e-5			
TENSTC	−4.460			
VV9	−1.035			

**Table 6:** The parameters used to predict hypertension in the reduced-parameter GLMs (“Renal”, “Liver” and “Renal+Liver”).

Name	Description	Unit	GLM
CPR	plasma protein concentration for protein destruction	g/L	Liver
AARK	basic afferent arteriolar resistance	mmHg min/L	Renal
EARK	basic efferent arteriolar resistance	mmHg min/L	Renal
LPPR	rate of liver protein production	g/min	Liver
GFLC	glomerular filtration coefficient	L/min/mmHg	Renal
NID	rate of sodium intake	mEq/min	Both

## Discussion

### Validity of the Guyton model

The Guyton model was constructed and refined over many years, and has been validated against a large amount of experimental data [23], [24]. However, many simplifications were necessary in order to permit simulated experiments under the computational resources that were available at the time [24], and the model does not incorporate recent advances in our understanding of the cardiovascular system. Thus, our results will tend to highlight the underlying assumptions and limitations of the Guyton model, rather than physiological phenomena. Indeed, one of the goals of this study was to provide sufficient data (in the supplementary material) to allow researchers to identify whether the Guyton model is sufficiently detailed for specific physiological applications. More recent models have incorporated greater levels of detail for individual organs [12], [74] or for the whole body [16], [19], and a comparison between the Guyton model and these newer models can illustrate the suitability of the Guyton model for clinical applications. Of course, the methodology we employed can be applied to these modern, more detailed models.

**Figure 9:**
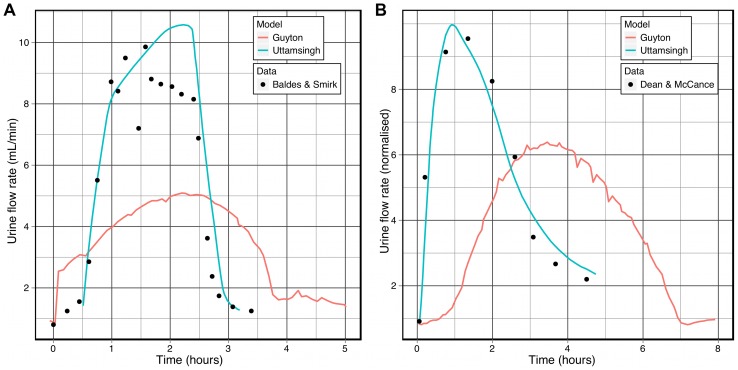
The effects of ingestion of hypotonic and hypertonic solutions on urine flow. These simulations reproduced the conditions shown in Figures 3 and 4 of Uttamsingh et al. [74], which include experimental from Baldes and Smirk [93] and Dean and McCance [75]. **A:** Urine flow following ingestion of 1 L of water. **B:** Urine flow following ingestion of hypertonic saline (normalized wrt. the urine flow rate prior to ingestion).

Here we present a brief comparison of the Guyton model to the human renal/body fluid model of Uttamsingh et al. [74], which was validated against several sets of experimental data. The result of ingestion of either hypotonic and hypertonic fluid in the Guyton model (shown in Figure 9) produces similar effects on the urine flow rate to that seen in the model of Uttamsingh et al. However, in response to the infusion of hypertonic saline (0.91 g of sodium chloride per kg of body weight, over a period of 65 minutes for a “normal human of 70 kg”) urine flow in the Guyton model increases at a slower rate, plateaus at a lower rate and eventually returns to the baseline level, while urine flow in [74] plateaus at twice the baseline and better matches the experimental data [75].

**Figure 10:**
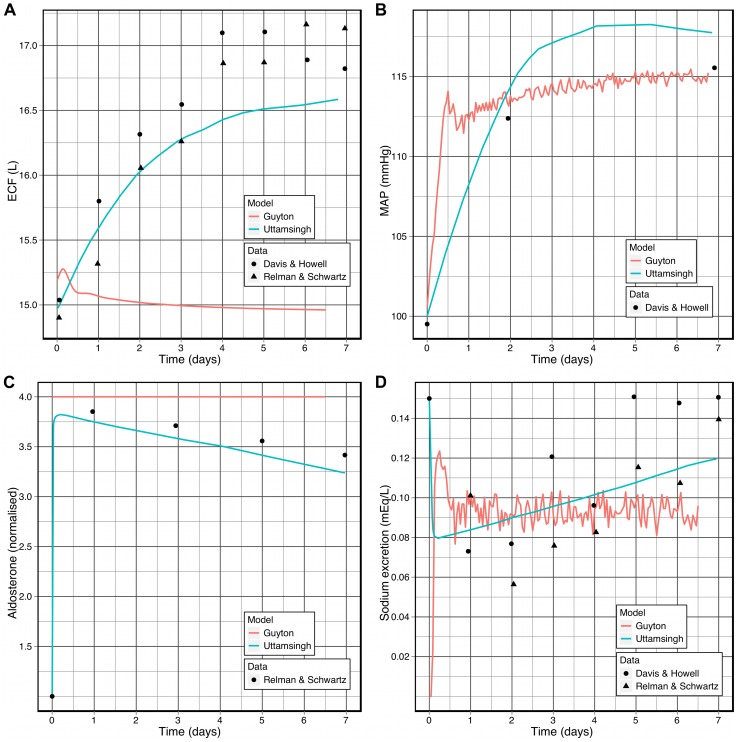
The effects of aldosterone loading on the human body. This simulation reproduced the conditions shown in Figure 5 of Uttamsingh et al. [74], which inclues experimental data from Davis and Howell [94] and Relman and Schwartz [76]. **A:** Extra-cellular fluid volume. **B:** Mean arterial pressure. **C:** Serum aldosterone (normalized). **D:** Sodium excretion rate.

Larger variation between the two models is observed when aldosterone is increased four-fold, in order to simulate the administration of deoxycorticosterone acetate (DOCA), a mineralocorticoid with similar effects to those of aldosterone [74]. The model of Uttamsingh et al. demonstrates gradual increases in extra-cellular fluid volume (1 L) and mean arterial pressure (10 mmHg), and a rapid drop in sodium excretion in response to the elevated aldosterone level, followed by a slow rise to match the rate of intake. The Guyton model, as shown in Figure 10, produces different behaviour. The extra-cellular fluid volume rises briefly and then gradually decreases until it is 0.1 L below the baseline (Figure 10a) and mean arterial pressure rapidly rises by 10 mmHg and then gradually increases by a further 2 mmHg (Figure 10b). Sodium excretion (Figure 10d) drops rapidly in the first 2 hours, then rises rapidly and overshoots in the following 6 hours, before equilibrating after 24 hours have elapsed. The Uttamsingh et al. model again matches the experimental data [76] better than the Guyton model (e. g., it reproduces the “escape” phenomenon, where the rate of sodium excretion eventually rises to match the increased rate of intake). However, the limited time-resolution (at most one data point every 24 hours) makes a precise comparison impossible. Indeed, with the exception of the extra-cellular fluid volume, the behaviour of the Guyton model also provides a reasonable fit to the data.

The differences highlighted here between the Guyton model and the model of Uttamsingh et al. are certainly due in part to the lower level of detail in the renal portion of the Guyton model, but the Guyton model also includes a more complete cardiovascular model, which would necessarily alter the dynamics produced in response to a chronic increase in aldosterone load. Thus, these observations *may* indicate a shortcoming in the Guyton model, but further analysis is required before a definitive statement can be made. These results highlight, however, the need to identify portions of the Guyton model that must be refined to replicate experimental data more recent than those used to originally validate the model. We discuss refinement of the Guyton model in the following section.

In our analysis we perturbed a single parameter in each simulaton (although each parameter was perturbed 1000 times, each simulation with a different set of randomly-selected parameter values). Perturbation of multiple parameters would yield a wealth of additional information, but without any guidance the only recourse would be to exhaustively search every combination of 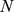 parameters, for 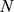 perturbations. Instead, with the results presented here one can select one parameter (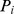) for perturbation and additionally perturb only those parameters that are significantly correlated with the effect of 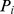 (as per our brief example: “Multi-parameter effects: accounting for the variance in HYL”).

### Application to individualized medicine

Given the population of virtual individuals that was presented here, an obvious and desirable application is to draw comparisons between subsets of this population and a given real-world patient. That is, given some observations of a real-world patient, we can select those virtual individuals who best match these observations and see whether one can draw conclusions about the condition of the real-world patient based on the long-term dynamics of the selected virtual individuals.

Beyond using virtual populations merely as a reference for the current and ongoing condition of real-world patients *who receive no intervention*, ongoing refinements of the Guyton model may ultimately support individualized health-care and individualized medicine. The application of mathematical models to individualized medicine would necessarily involve integrating detailed models of physiology, pharmacokinetics and pharmacodynamics. Current efforts on this front include the BIMBO project [77].

Development of chronic diseases such as cardiovascular disease is a complex process that involves environmental and cultural factors shared by the individuals living in the same geographical area, as well as ageing, genetic and disease determinants. Hunter et al. [3] have emphasized the need for diagnostic workflows on the prediction of risk that integrates the influence of both population and patient-specific information in support of tailored interventions aiming at optimizing diagnosis and treatment planning and monitoring.

Researchers of the BIMBO project have defined a modeling approach to estimate the public health impact, in terms of the reduction in the number of cardiovascular deaths (CVD), of administering blood pressure lowering drugs to a virtual population of patients [77]. That virtual population [77] (distinct from the virtual population presented here) reproduces the demographic composition as well as the cardiovascular risk factor profiles of a country population, each virtual individual being characterized by a number of features allowing estimation of CVD risk and treatment efficacy. The individuals eligible for treatment could be selected from their computed CVD risk over a fixed threshold and by having blood pressure in excess of 140/90 mmHg. The authors used a simplified approach where treatment effect was represented by the relative risk, which was assumed to be constant over time and among different individuals, to estimate the public health impact of BP lowering drugs [77].

The work presented here illustrates the value of using population information to predict the success of treatment strategies, whilst also moving towards a more ambitious goal: taking into account the individual genetic backgrounds and pathophysiological profiles. This would contribute to the delivery of individualized healthcare, by optimizing the impact of treatments for both the individual patient and at the population level. Future challenges include the development of more sophisticated effect models [78], such that relative-risks and odds ratios depend on individual characteristics which affect the pharmacokinetic and/or pharmacodynamic parts of the model [79]. Realization of these goals would represent a significant step towards personalizing anti-hypertensive treatment.

The implications of pharmacogenetic parameters on drug efficacy have been explored in the context of diuretic treatment for blood pressure [80]–[82]. One candidate for the identification of responders to thiazide diuretics is the polymorphic gene coding the cytoskeleton protein 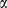-adducin, whose mutant form has been associated with an increased rate of sodium reabsorption [83], [84], elevated blood pressure [85], [86], salt-sensitivity [87] and increased risk of cardiovascular events [88]. The same associations first documented in Caucasian populations [84], [87] have not been reported in all other populations, with contradictory evidence from studies in Chinese, African American and Japanese populations [89], suggesting the role of additional factors in mediating the effects attributed to the 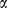-adducin polymorphism. But before rejecting the hypothesis of a pharmacogenetic effect of the 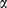-adducin variant, a number of epistatic interactions and environmental influences contained in the virtual population characteristics (e. g., different degrees of RAAS activation in response to salt consumption) could be explored through physiological modeling.

With regard to the diagnosis and treatment of hypertension, a practical model would predict the effects of the various diuretics and other drugs that are commonly administered to ameliorate hypertension. This would allow the model predictions to be directly compared to clinical studies such as INDANA [90]. To this end, refinements are being incorporated into the original Guyton model [32] as part of the SAPHIR project [13], such as a detailed model of the RAAS [34]. The culmination of these efforts will result in a richly-detailed and more accurate model of renal autoregulation being incorporated into the Guyton model, providing a platform for pharmacological predictions that may assist in the diagnosis and treatment of hypertension [77].

### Conclusion

We have presented a sensitivity analysis of the Guyton model of human physiology (1992 version), which examined the elementary effects of each parameter over a range of timescales and the correlations between model parameters and key output variables. We also demonstrated how interesting multi-parameter combinations can be identified, and how this can highlight shortcomings in the model.

A pool of 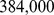 simulations with randomized parameters (analagous to genetic variants) was generated for this analysis, forming a diverse *virtual population* of 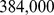 virtual individuals from which representative subsets can be drawn to match characteristics of individual real-world patients. The population was divided into normotensive and hypertensive sub-populations, and a 6-parameter linear classifier was shown to have good predictive power for identifying hypertensive virtual individuals, based on parameters that are feasible to estimate *in vivo*.

Work is currently underway on comparing these results to real-world patient data from clinical studies of the effect of Avastin on hypertension in cancer patients [91], [92]. About half of the patients develop hypertension in response to Avastin, and are also the most likely to experience a remission. The analysis will aim to identify whether any of the elementary effects or correlations presented in this manuscript are evident in real-world patients, and to evaluate the use of the virtual population in selecting regions of the parameter space of the Guyton model that correspond to the characteristics of a real-world patient. This exploratory project is at a preliminary stage and no results can be presented at this time.

The methodology we have presented here and applied to the Guyton model is generic in that it can be applied to any mathematical model of sufficient complexity. As physiological models encompass larger and larger scales, both spatially and temporally, this methodology should prove beneficial in elucidating the subtle interactions between model parameters in these complex models.

Such an effort is required to evaluate the clinical suitability of using the Guyton model to assist in providing individualized predictive medicine, as per the goals of both the IUPS Physiome and the Virtual Physiological Human projects.

## Supporting Information

Dataset S1 **Simulation results, elementary effects and correlations.** This dataset contains the results of each individual simulation, the calculated elementary effects, correlations between each parameter and variable, and correlations between elementary effects and parameters. Dryad Digital Repository. http://dx.doi.org/10.5061/dryad.h3s0r.
(ZIP)

Table S1 **Descriptions of the 96 model parameters included in the sensitivity analysis.** The distribution of these 96 parameters was: 32 cardiac, 21 renal, 16 autoregulation, 16 hormonal, 11 local circulation, and 4 thirst-related.
(PDF)

Table S2 **Descriptions of the 276 output variables included in the sensitivity analysis.**
(PDF)
