# Supplementary material for: Virtual Patients and Sensitivity Analysis of the Guyton Model of Blood Pressure Regulation: Towards Individualized Models of Whole-Body Physiology
Source: PLoS Comput Biol. 2012 Jun 28;8(6):e1002571. doi: 10.1371/journal.pcbi.1002571 (PMC3386164; doi:10.1371/journal.pcbi.1002571)
Supplement: Table S1 — Descriptions of the 96 model parameters included in the sensitivity analysis. The distribution of these 96 parameters was: 32 cardiac, 21 renal, 16 autoregulation, 16 hormonal, 11 local circulation, and 4 thirst-related. (PDF) [file pcbi.1002571.s002.pdf]

Table S1. Descriptions of the 96 model parameters that were included in the sensitivity analysis, and their minimum and maximum values. The distribution of these 96 parameters was: 32 cardiac, 21 renal, 16 autoregulation, 16 hormonal, 11 local circulation, and 4 thirst-related.

Name	Description	Unit	Min	Max
A1K	time constant, rapid nonmuscle autoregulation	min	0.25	1.0
A2K	time constant, intermediate nonmuscle autoregulation	min	30.0	120.0
A3K	time constant, long-term nonmuscle autoregulation	min	24000	80000.0
A4K	time constant, rapid muscle autoregulation	min	0.05	0.2
A4K2	time constant, long-term muscle autoregulation	min	20000.0	80000.0
AARK	basic afferent arteriolar resistance	mmHg min/L	0.62	1.2
ADHPAM	sensitivity control of arterial pressure on ADH	–	0.15	0.6
ADHTC	time constant, ADH secretion	min	7.5	30.0
AH11	time constant, volume receptor adaptation	min	500.0	2000.0
AH9	sensitivity control of AHZ	–	0.5	2.0
AHMNAR	sensitivity control, renal effect of ADHMK	–	0.15	0.6
AHTHM	sensitivity controller, effect of ADH on thirst	–	1.0	4.0
ALCLK	sensitivity controller of AMK1	–	0.15	0.6
ALDMM	sensitivity controller of aldosterone multiplier	–	1.25	5.0
AMCSNS	sensitivity controller, aldosterone secretion	–	0.4	1.0
AMKM	sensitivity controller of AMK	–	0.3	0.8
AMKMUL	sensitivity controller of AMR	–	6.0	24.0
AMNAM	sensitivity controller of AMNA	–	0.4	1.6
AMT	time constant, aldo accumulation and destruction	min	40	100.0
ANCSNS	sensitivity controller of ANM	–	0.23	0.8
ANMALD	sensitivity controller, angiotensin effect on aldo secretion	–	1.25	5.0
ANMAM	sensitivity effect of angiotensin on afferent arterioles	–	1.0	1.688
ANMEM	sensitivity effect of angiotensin on efferent arterioles	–	1.0	2.0
ANMKEM	sensitivity controller of ANMKE	–	1.0	4.0
ANMNAM	sensitivity controller of DTNANG	–	0.5	2.0
ANMSLT	sensitivity controller ANMSML	–	1.0	4.0
ANMTM	sensitivity controller of ANMTH	–	0.75	3.0
ANPTC	time constant, ANP accumulation and destruction	min	2.0	8.0
ANPXAF	sensitivity factor of ANP on renal afferent resistance	–	0.5	4.0
ANT	time constant, angiotensin accumulation and destruction	min	6.0	24.0
ANUM	sensitivity controller of ANU	–	3.0	8.0
ANV	time constant, angiotensin secretion	min	2500.0	10000.0
ANY	sensitivity controller of ANU for venous volume	–	-0.4	-0.15
AUC1	sensitivity controller of AUC	–	0.15	0.6
AUK	time constant of baroreceptor adaptation	min	0.002	0.008
AUMK1	autonomic sensitivity controller on EAR	–	0.15	0.6
AUN1	sensitivity controller of AUN	–	0.25	1.0
AUS	sensitivity of sympathetic control of heart rate	–	0.333	4.0
AUTOK	rate of development of very rapid autoregulation	–	2.33	28.0
AUTOSN	overall sensitivity controller, nonmuscle autoreg.	–	0.0333	0.4
AUV	blood volume shifted from unstressed to stressed	–	0.183	2.2
AUX	sensitivity controller of arterial baroreceptors	–	0.5	2.0

BAROTC	time constant, baroreceptor (AU6)	min	0.08	0.32
CFC	capillary filtration coefficient	mmHg min/L	0.0039	0.02
CNR	reference sodium conc to determine ADH secretion rate	mEq/L	130.0	141.9
CPF	pulmonary capillary filtration coefficient	–	0.0001	0.0012
CPR	critical plasma protein conc for protein destruction	g/L	23.0	62.0
CV	modified venous compliance	L/mmHg	0.0333	0.16
DHDTR	proportionality constant, cardiac deterioration	–	0.0167	0.2
DIURET	diuretic effect on tubular reabsorption	–	0.58	4.0
DTNAR	sensitivity controller of DTNARA	–	0.3375	1.05
EARK	basic efferent arteriolar resistance	mmHg min/L	0.67	1.5
GFLC	glomerular filtration coefficient	L/min	0.00693	0.026
HM6	erythropoietic limiter	mmHg	617.0	2700.0
HM8	sensitivity control for erythropoiesis	–	4.57×10^{-8}	5×10^{-8}
HSL	basic strength of left ventricle	–	0.58	1.6
HSR	basic strength of right ventricle	–	0.6	1.42
HTAUML	autonomic sensitivity on PRA	–	0.133	0.9
HYL	quantity of hyaluronic acid in tissues	mEq	40.0	75.0
KID	rate of potassium intake	mEq/min	0.0267	0.16
KORGN	gain of positive feedback, korner concept	–	0.333	4.0
KORTC	time constant, korner effect	min	5000.0	20000.0
LPPR	rate of liver protein production	g/min	0.01	0.12
MDFLKM	sensitivity controller of MDFLK	–	0.3335	1.26
MDFLWX	sensitivity controller of MDFL3	–	0.005	0.02
NID	rate of sodium intake	mEq/min	0.04	0.25
O2A	sensitivity controller of AOM, autonomic on metabolism	–	0.05	0.2
O2CHMO	O_2 chemoreceptors sensitivity controller	–	0.005	0.02
O2M	basic O_2 utilization in nonmuscle tissues	L/min	138.0	180.0
OMM	basic O_2 utilization in muscle tissues (at rest)	L/min	39.0	95.0
PCR	critical capillary pressure for protein leakage	mmHg	5.0	20.0
PM5	constant, to change muscle capillarity	–	20.0	120.0
POR	reference value of capillary PO_2 in non-muscle tissue	mmHg	25.0	41.0
QRF	left ventricle funct effect on right ventricle function	–	0.05	0.6
RABSC	peritubular capillary reabsorption coefficient	–	0.167	0.8
RFABDM	sensitivity controller of RFABD	–	0.15	0.4
RFABKM	proportionality constant, RFABK	–	0.01	0.053
RNAGTC	time constant, renal autoregulation feedback	min	7.5	30.0
RNAUGN	basal renal autoregulation feedback multiplier	–	0.2	1.1
RTPPR	renal oncotic, internal var	–	0.7	3.6
RTPPRS	renal oncotic, internal var	mmHg	5.07	18.0
RTSPRS	renal tissue fluid pressure	mmHg	3.0	10.0
RVSM	basal systemic venous multiplier	–	0.7	1.5
SR	sensitivity controller, short-term stress relaxation	–	0.5	2.0
SR2	sensitivity controller, long-term stress relaxation	–	0.5	2.0
SRK	short time constant for stress relaxation	min	2.5	10.0
SRK2	long time constant for stress relaxation	min	5000.0	20000.0
TENSTC	time constant, myogenic autoregulation	min	0.025	0.1
TSSLML	sensitivity controller of VTS	–	0.075	0.3
TSSLTC	VTS1 time constant	min	0.0025	0.01
TVDDL	TVD damping coefficient	–	10.0	90.0
U	damping coefficient, circulation	–	0.08	0.96
VIDML	proportionality constant, CCD to VID	–	0.02	0.05
VNTSTM	sensitivity controller for alveolar ventilation	–	0.5	2.0
VPTISS	pulmonary tissue fluid volume	L	0.00583	0.07
VV9	basic venous volume, without autonomic drive	L	2.2	2.7
