# Supplementary material for: Virtual Patients and Sensitivity Analysis of the Guyton Model of Blood Pressure Regulation: Towards Individualized Models of Whole-Body Physiology
Source: PLoS Comput Biol. 2012 Jun 28;8(6):e1002571. doi: 10.1371/journal.pcbi.1002571 (PMC3386164; doi:10.1371/journal.pcbi.1002571)
Supplement: Table S2 — Descriptions of the 276 output variables included in the sensitivity analysis. (PDF) [file pcbi.1002571.s003.pdf]

Table S2. Descriptions of the 276 output variables that were included in the sensitivity analysis.

Name	Description	Unit
A1B	arterial baroreceptor drive	–
AAR	afferent arteriolar resistance	mmHg min/L
ADH	total ADH secretion (pressure, sodium, infusion)	–
ADHC	ADH concentration	mEq/L
ADHMK	effect of ADH on sodium and water reabsorption	–
ADHMV	effect of ADH on nonrenal vascular resistance	–
ADHNA	effect of plasma sodium on ADH secretion	–
ADHPA	effect of arterial pressure on ADH secretion	mmHg
ADHPR	arterial pressure effect on ADH secretion	–
ADHZ	internal variable for ADH concentration	–
AH7	volume receptor output	–
AHY	adapted effect, volume receptor output	–
AHZ	basic effect, on volume receptor output	–
ALVENT	alveolar ventilation	L
AM	general aldosterone multiplier, ratio to normal	–
AM1	rate of aldosterone secretion	mEq/min
AMC	aldosterone concentration, ratio to normal	–
AMK	effect of aldosterone on cell membrane potassium transport	–
AMK1	effect of aldosterone on cell membrane potassium transport	–
AMM	overall multiplier factor for muscle autoregulation	–
AMM1	multiplier for rapid muscle autoregulation	–
AMM2	multiplier for long-term muscle autoregulation	–
AMNA	aldosterone effect on tubular Na reasorption	–
AMR	aldosterone secretion, ratio to normal conc.	–
AMR1	total aldosterone secretion (endo + exo)	mEq/min
AMRBSC	basal aldo secretion due to angiotensin and potassium	mEq/min
ANC	plasma angiotensin concentration	mEq/L
ANGSCR	angiotensin secretion due to macula densa flow	mEq/min
ANM	general angiotensin multiplier effect, ratio to normal	–
ANMAR	angiotension multiplier on afferent arterioles	–
ANMER	angiotension multiplier on efferent arterioles	–
ANMKE	angiotensin effect on renal potassium reabsorption	–
ANP	rate of endogenous ANP secretion	mEq/min
ANP1	total rate of ANP secretion (endo + exo)	mEq/min
ANPC	plasma ANP concentration	mEq/L
ANPL	left atrial ANP secretion	mEq/min
ANPR	rate of endogenous ANP secretion	mEq/min
ANPR1	total rate of ANP secretion (endo + exo)	mEq/min
ANPR2	right atrial ANP secretion	mEq/min
ANPX	ANP multiplier, ratio to normal	–
ANU	angiotensin effect on arterial resist + venous volume	–
ANUVN	effect of angiotensin on systemic veins	–
ANX	angiotensin secretion	mEq/min
ANX1	actual ANX after damping	mEq/min
AOM	autonomic effect on tissue oxygen utilization	–
APD	pressure drop in afferent arterioles	mmHg
AR1	multiplier for rapid non-muscle autoregulation	–
AR2	multiplier for intermediate non-muscle autoregulation	–
AR3	multiplier for long-term non-muscle autoregulation	–
ARM	non-muscle global autoregulation multiplier	–

ATRRFB	volume receptor feedback on arterial resistance	–
ATRVFB	volume receptor feedback on unstressed venous volume	–
AU	overall activity of autonomic system	–
AU1	integral of DAU	–
AU2	internal variable, baroreceptor adaptation	–
AU4	long-term baroreceptor adaptation output	–
AU6	baroreceptor output	–
AU8	rate of change of baroreceptor output, adapted	–
AUB	arterial baroreceptor drive, before damping-adaptation	–
AUC	effect of chemoreceptors on autonomic stimulation	–
AUC2	autonomic drive due to hypoxemia (for AUTTL)	–
AUH	autonomic effect on heart strength, ratio to normal	–
AULP	autonomic drive due to low pressure receptors	–
AUM	sympathetic vasoconstrictor effect on arteries	–
AUMK	autonomic multiplier factor on renal vessels	–
AUN	effect of CNS ischemic reflex on autoregulation	–
AUO	delta of overall activity of autonomic system	–
AUR	autonomic stimulation of heart rate	–
AUTTL	internal variable, overall autonomic output	–
AVE	autonomic effect on venous resistance	–
BFM	muscle blood flow	L/min
BFN	nonmuscle nonrenal blood flow	L/min
CCD	electrolyte gradient across cell membrane	mEq/L
CHY	hyaluronic acid conc. in tissue fluids	mEq/L
CKE	extracellular potassium concentration	mEq/L
CKI	intracellular potassium concentration	mEq/L
CN3	venous stretch component due to capillary pressure	–
CNA	extracellular sodium concentration	mEq/L
CPA	pulmonary artery pressure effect on pulm. resistance	L/(min mmHg)
CPI	free interstitial fluid protein conc.	g/L
CPN	protein conc. in pulmonary fluids	g/L
CPP	plasma protein concentration	g/L
CPPD	error signal for liver protein destruction	g/L
DAS	rate of change of blood volume in the aorta	L/min
DAU	rate of change, autonomic drive	min^{-1}
DFP	rate of change in pulmonary free fluid volume	L/min
DFZ	DFP before damping	L/min
DHM	rate of cardiac deterioration caused by hypoxia	$\text{min}^{-}1$
DLA	rate of change of blood volume in left atrium	L/min
DLP	net rate of liver protein production	g/min
DO2M	rate of change of oxygen in muscle tissue	L/min
DO2N	rate of change of oxygen in nonmuscle tissue	L/min
DO2VAD	change in ventilatory response (due to long tc)	L/min
DOB	rate of oxygen delivery to non-muscle cells	L/min
DOVA	rate of change in arterial O_2 content	–
DPA	rate of change in pulmonary blood volume	L/min
DPC	loss of plasma proteins through systemic capillaries	g/min
DPI	change of protein in free interstitial fluid	g/min
DPL	rate of systemic lymphatic return of protein	g/min
DPP	rate of change of plasma protein	g/min
DRA	rate of change of blood volume in right atrium	L/min
DTKA	distal reabsorption of potassium	mEq/min
DTKI	distal delivery of potassium	mEq/min
DTKSC	distal secretion of potassium	mEq/min

DTNAI	distal tubular sodium delivery	mEq/min
DTNANG	rate of distal Na reabsorption due to angiotensin	mEq/min
DTNARA	rate of distal tubular Na reabsorption (non ang-II)	mEq/min
DTURI	rate of urinary urea excretion	mEq/min
DVS	rate of change of venous volume	L/min
EAR	efferent arteriolar resistance	mmHg min/L
EFAFPR	total to efferent renal plasma flow fraction	–
FISFLO	fistula blood flow	L/min
GFR	glomerular filtration rate	L/min
GLP	glomerular pressure	mmHg
GLPC	glomerular colloid osmotic pressure	mmHg
HM	hematocrit (%)	–
HM1	hematocrit	–
HM3	erythropoietic effect of PO_2 (low PO_2)	mmHg
HM4	erythropoietic effect of PO_2	mmHg
HM5	sum of HM3 and HM4	mmHg
HM7	final erythropoietic effect of PO_2	mmHg
HMD	cardiac depressant effect of hypoxia	–
HPEF	pumping effectiveness of the right heart	–
HPL	hypertrophy effect on left ventricle	–
HPR	hypertrophy effect on right ventricle	–
HR	heart rate (beats/min)	min^{-1}
KE	total extracellular fluid potassium	mEq
KI	total intracellular fluid potassium	mEq
KOD	rate of renal potassium excretion	mEq/min
KODN	rate of potassium excretion, normal kidneys	mEq/min
KTOT	total body potassium (intra + extracellular)	mEq
KTOTD	rate of change of total body potassium	mEq/min
MAP	systemic arterial pressure (PA)	mmHg
MDFLK	macula densa flow, potassium component	–
MDFLW	normalized tubular flow at macula densa	–
MDFLW3	macula densa flow effect on renin release	–
MMO	rate of oxygen utilization by muscle cells	L/min
MO2	rate of oxygen utilization by non-muscle cells	L/min
MYOGRS	myogenic autoregulation multiplier	–
NAE	total extracellular sodium	mEq
NED	rate of change of extracellular sodium	mEq/min
NOD	rate of renal excretion of sodium	mEq/min
NODN	rate of sodium excretion, normal kidneys	mEq/min
O2DFS	pulmonary O_2 diffusion	L/min
O2UTIL	total rate of O_2 delivery (muscle & nonmuscle)	L/min
O2VAD1	delayed ventilatory response	L
O2VAD2	delayed ventilatory response	L
O2VTS2	reciprocal of O2VTST	L^{-1}
O2VTST	acute ventilatory response	L
OSA	aortic oxygen saturation	–
OSMOPN	urinary osmolar excretion	mEq/min
OSV	nonmuscle venous oxygen saturation	–
OVA	arterial O_2 content	–
OVS	muscle venous oxygen saturation	–
P1O	effective nonmuscle PO_2 (from POT)	mmHg
P2O	effective muscle PO_2 (from PMO)	mmHg
PA	systemic arterial pressure (MAP)	mmHg
PA1	effective arterial pressure driving autonomic system	mmHg

PA2	effective arterial pressure driving left ventricle pumping	mmHg
PAM	pressure effect on arterial distension, ratio to normal	–
PAMK	korner pressure effect on non-renal vessels	–
PAMKRN	korner pressure effect on renal vessels	–
PAR	renal arterial pressure	mmHg
PC	capillary pressure	mmHg
PCP	pulmonary capillary pressure	mmHg
PDO	driving PO_2 for muscle autoregulation	mmHg
PFI	transfer of fluid across pulmonary capillaries	L/min
PFL	renal filtration pressure	mmHg
PGH	hydrostatic pressure in tissue gel	mmHg
PGL	pressure gradient in lungs	mmHg
PGS	pressure gradient from arteries to veins	mmHg
PIF	interstitial fluid pressure	mmHg
PL1	internal variable, effect of PLA on RPV	–
PLA	left atrial pressure	mmHg
PLA1	effective left atrial press. effect on cardiac output	mmHg
PLD	pressure gradient to cause lymphatic flow	mmHg
PLF	pulmonary lymphatic flow	L/min
PLUR	total body urea	mEq
PLURC	plasma urea concentration	mEq/L
PMC	mean circulatory filling pressure	mmHg
PMO	muscle tissue PO_2	mmHg
PMP	mean pulmonary filling pressure	mmHg
PMS	mean systemic filling pressure	mmHg
PO2ALV	alveolar PO_2	mmHg
PO2AM1	effective ambient PO_2 on HM3	mmHg
PO2ART	arterial PO_2	mmHg
POA	vasoconstrictor effect of intermediate autoregulation	–
POB	vasoconstrictor effect of rapid autoregulation	–
POC	vasoconstrictor effect of long-term autoregulation	–
POD	non-muscle PO_2 (minus normal value)	mmHg
POE	driving pressure for rapid muscle autoregulation	mmHg
POS	pulmonary interstitial fluid colloid osmotic pressure	mmHg
POSHYL	tissue osmotic pressure due to hyaluronic acid	mmHg
POT	non-muscle tissue PO_2	mmHg
POV	non-muscle venous PO_2	mmHg
PP1	relates pulm. art pressure to pulm. art resistance	–
PP2	effective pulm. artery pressure on right ventr. pumping	mmHg
PPA	pulmonary arterial pressure	mmHg
PPC	plasma colloid osmotic pressure	mmHg
PPD	rate of change of protein in pulmonary fluids	g/min
PPI	pulmonary interstitial fluid pressure	mmHg
PPN	rate of pulmonary capillary protein loss	g/min
PPO	pulmonary lymph protein flow	g/min
PPR	total protein in pulmonary fluids	g
PPZ	PPD before damping	g/min
PR1	outflow pressure from the veins into the chest	mmHg
PRA	right atrial pressure	mmHg
PRA1	effective right atrial pressure	mmHg
PRCD	capillary pressure gradient for protein leakage	mmHg
PRP	total plasma protein	g
PTC	total osmotic pressure of tissue gel	mmHg
PTCPR	interstitial fluid colloid osmotic pressure (protein)	mmHg

PTS	interstitial solid tissue pressure	mmHg
PTT	total tissue pressure	mmHg
PVO	muscle venous PO_2	mmHg
PVS	average pressure in major veins	mmHg
QAO	aortic blood flow (cardiac output)	L/min
QLN	basic left ventricular output	L/min
QLO	left ventricular output	L/min
QLO1	passive left ventricular output	L/min
QO2	quantity of O_2 in nonmuscle tissue	L
QOM	quantity of O_2 in muscle tissue	L
QPO	blood flow into pulmonary veins and left atrium	L/min
QRN	basic right ventricular output	L/min
QRO	actual right ventricular output	L/min
QVO	rate of blood flow from veins into right atrium	L/min
R1	composite multiplier for non-renal resistance	–
RABSPR	starling pressure gradient for proximal reabsorption	mmHg
RAD	myogenic autoregulation	–
RBF	renal blood flow	L/min
RC1	rate of red cell production	L/min
RC2	rate of red cell destruction	L/min
RCD	rate of change of red cell mass	L/min
RCPRS	renal peritubular capillary pressure	mmHg
RFAB	RFAB1 after damping	L/min
RFAB1	renal peritubular capillary reabsorption	L/min
RFABK	effect of physical forces on potassium reabsorption	mEq/min
RFN	renal blood flow, undamaged kidney	L/min
RMO	rate of O_2 delivery to muscle tissues	L/min
RMULT1	very rapid autoregulation multiplier	–
RNAUG1	autoregulatory feedback multiplier on AAR	–
RNAUG2	total autoregulatory feedback multiplier on AAR	–
RNAUG3	autoregulatory feedback on AAR due to adaptation	–
RPA	pulmonary arterial resistance	mmHg min/L
RPT	total pulmonary vascular resistance	mmHg min/L
RPV	pulmonary venous resistance	mmHg min/L
RR	total renal resistance	mmHg min/L
RSM	vascular resistance through muscle tissues	mmHg min/L
RSN	vascular resistance through non-muscle tissues (non-renal)	mmHg min/L
RSPDFC	respiratory O_2 diffusion coefficient	mmHg min/L
RTP	total peripheral resistance	mmHg min/L
RTSPPC	renal tissue fluid oncotic pressure	mmHg
RV1	multiplier effect of vascular stretch on venous resistance	–
RVG	resistance from veins to right atrium	mmHg min/L
RVM	pulmonary pressure load on right ventricle	L/min
RVS	resistance to blood flow through the veins	mmHg min/L
STH	effect of hypoxia + angiotensin on salt and water intake	–
SVO	stroke volume output	L
SYSFLO	systemic blood flow	L/min
TSP	total interstitial tissue protein	g
TVD	rate of drinking	L/min
UROD	rate of urinary urea excretion	mEq/min
VAE	excess volume in the arterial tree	L
VAS	volume in systemic arteries	L
VB	blood volume	L
VBD	change in blood volume distributed in the circulation	L

VEC	extracellular fluid volume	L
VIB	blood viscosity, ratio to that of water	–
VIC	intracellular fluid volume	L
VID	rate of fluid transfer across cell membrane	L/min
VIE	portion of blood viscosity caused by red blood cells	–
VIF	volume of free interstitial fluid	L
VIM	blood viscosity effect on resistance, ratio to normal	–
VLA	volume of left atrium	L
VLE	excess blood volume in left atrium	L
VP	plasma volume	L
VPA	volume in pulmonary arteries	L
VPD	rate of change in plasma volume	L/min
VPE	excess blood volume in pulmonary tree	L
VPF	pulmonary free fluid volume	L
VRA	volume of right atrium	L
VRC	volume of red blood cells	L
VRE	volume of right atrium in excess	L
VTC	rate of fluid transfer across systemic capillaries	L/min
VTCPL	rate of plasma leakage across systemic capillaries	L/min
VTL	rate of systemic lymph flow	L/min
VTS	systemic interstitial fluid volume	L
VTS1	VTS after damping	L
VTS2	VTS component due to interst. stress relaxation	L
VTW	total body water	L
VUD	actual rate of urinary output	L/min
VUDN	rate of urinary output, normal kidneys	L/min
VV6	vascular volume caused by long-term stress relaxation	L
VV7	vascular volume caused by short-term stress relaxation	L
VVE	excess volume of blood in the veins	L
VVR	basic venous volume (all other factors=normal)	L
VVS	actual venous vascular volume	L
